# Antiretroviral Therapy Initiation Alters the Redox System of Asymptomatic HIV-Infected Individuals: A Longitudinal Study

**DOI:** 10.1155/2017/9834803

**Published:** 2017-03-21

**Authors:** Karen Ingrid Tasca, Juliana Trindade Caleffi, Camila Renata Correa, Mariana Gatto, Francilene Capel Tavares, Caio Cavassan Camargo, Alexandrina Sartori, Mara Biasin, Lenice do Rosário de Souza

**Affiliations:** ^1^Department of Tropical Diseases, Botucatu Medical School (FMB), Universidade Estadual Paulista (UNESP), Botucatu, SP, Brazil; ^2^Department of Pathology, FMB, UNESP, Botucatu, SP, Brazil; ^3^Department of Biomedical and Clinical Sciences, University of Milan, Milan, Italy

## Abstract

*Background*. The combination antiretroviral therapy (cART) increases the oxidative stress in HIV-infected people, which in turn favors the onset and aggravation of non-AIDS comorbidities, a common situation affecting these individuals. We aimed to evaluate the influence of cART initiation on oxidative stress parameters. This is a longitudinal study including 30 asymptomatic patients divided according to their CD4+ T cell count (G1: <500 cell/mL; G2: >500 cell/mL) before (M0) and after (M1) cART initiation. We analyzed total antioxidant capacity (TAC), fat-soluble vitamins, malondialdehyde, 8-isoprostane, and DNA damage.* Results*. Results showed a decrease in TAC, retinol, *α*-tocopherol, and some carotenoids, in addition to a significant increase in DNA damage at M1. These changes were more evident in G2 subjects. Moreover, there was a significant 8-isoprostane increase at M1 in individuals belonging to G1.* Conclusion*. The results indicate that cART interfered in the redox system, mainly by reducing the antioxidant defenses. In addition, patients who had CD4+ T counts higher than 500 cells/mm^3^ showed more susceptibility to genotoxicity, while patients with less CD4+ T counts displayed more damage triggered by lipoperoxidation. Considering the early beginning of cART, its chronic use, and its capacity to alter the redox status, further long-term studies on larger cohorts are needed to define the best time to initiate therapy and to investigate new strategies to delay the development of non-AIDS diseases.

## 1. Introduction

Nowadays, there is increasingly early recommendation for therapeutic beginning in HIV-infected people, a strategy to promote benefits in patients and to reduce transmission rates [1–3]. However, due to prolonged use of antiretroviral therapy (cART), its toxic effects should be taken into account. In addition to kidney, liver, and bone impairment, many drugs are responsible for the onset of the metabolic syndrome and consequently for the increase of non-AIDS comorbidities, what could be contributing to current causes of death in this population such as cardiovascular disease and diabetes mellitus, among others [1, 4–6].

cART also seems to contribute to the imbalance between oxidants and antioxidants agents in people living with HIV/AIDS (PLWHA) by increasing the former and decreasing the latter [6–9]. Such oxidative stress results in dysregulation of signaling and redox control, possibly leading to molecular damage [10]. As a result of this process, genotoxic and mutational effects contributing to the development of cancer and other non-AIDS diseases may occur [11, 12], and such homeostatic instability could even accelerate the progression of HIV infection to AIDS [12], even this relationship being speculative.

The literature points out that lipid peroxidation, as measured, for example, by serum analysis of malondialdehyde (MDA) [13, 14] and 8-isoprostanes [6, 15], is higher in PLWHA, compared to noninfected individuals [13, 14], and it increases following cART initiation [6, 15]. In addition, a recent study with these markers has shown that oxidative stress was a predictor of all-cause mortality in HIV-infected patients, and, for F2-isoprostane, this association is independent of HIV-related factors and subclinical inflammation [16]. Further, HIV-individuals under cART have less total antioxidant capacity (TAC) [17] and a lower vitamin profile [8, 18] as compared to patients naïve to treatment. Thus not only the pathogenesis of HIV but also therapy may influence the increase in prooxidants and the decrease in antioxidants in PLWHA, leading to consequent damage to important biomolecules as lipids, protein, and nucleic acids [9, 19, 20].

For this reason we hypothesized that cART can increase the oxidative status of PLWHA already in the initial months of treatment, which could occur differently in patients, according to their pre-cART CD4+ T counts.

The objective of this study was to analyze the influence of cART initiation on oxidative stress parameters in asymptomatic HIV-infected individuals.

Compared to previous studies that have already investigated some of these markers, this study will take into consideration all of these parameters in the same cohort, in a strictly controlled population (without comorbidities, coinfections, or habits that could influence the marker levels) and relative to different CD4+ T cells before therapeutic onset. Moreover to our knowledge, no previous studies investigated the nuclear DNA damage by comet test in this population under the given conditions.

## 2. Material and Methods

### 2.1. Study Design

This longitudinal study was conducted between 2012 and 2015, on 30 HIV+ patients who attended at the Specialist Outpatient Service for Infectious Diseases “Domingos Alves Meira,” at the Botucatu Medical School Complex (FMB)-UNESP, in São Paulo state, Brazil. This Service covers about 600 HIV-infected people from Botucatu and surrounding area; for this study, 150 consecutive antiretroviral treatment-naïve patients were interviewed, but only 30 of them were included according to the exclusion criteria.

Data collection and biological samples were carried out in two stages: before therapy initiation (M0) and approximately eight months after that intervention (M1), when the second medical appointment was conveniently scheduled and comprised between 30 and 48 weeks. This second period of analysis was given appropriate, considering the high undetectability rates observed in our service combined with the data presented in the “Guidelines for the Use of Antiretroviral Agents in HIV-1-Infected Adults and Adolescents-2014”: “Individuals who are adherent to their ARV regimens and do not harbor resistance mutations to the component drugs can generally achieve viral suppression 8 to 24 weeks after ART initiation; rarely, in some patients it may take longer” [21].

In Brazil, as well as in some other countries, the current recommendation by the Ministry of Health is that cART should be offered to all HIV-infected patients, regardless of CD4+ T counts [2]. However, until December 2013, the treatment was indicated only for asymptomatic individuals with CD4+ T counts below 500 cells/mL. Therefore, in our study, patients were also divided according to their pre-cART CD4+ T cell count, (G1: <500 cells/mL; G2: >500 cells/mL).

### 2.2. Inclusion and Exclusion Criteria

PLWHA inclusion criteria were age comprised between 20 and 50 years old, no previous cART administration, and signing an Informed Consent Form.

Considering many habits and comorbidities could be confounding variables and would interfere in our analysis of oxidative stress [22], patients carrying any of the following conditions were excluded: use of vitamin supplements, cancer history (current or previous), anorexia, morbid obesity, diabetes* mellitus*, cardiovascular, genetic or autoimmune diseases, organ transplants, use of illicit drugs and alcohol, pregnancy at any stage or breastfeeding, AIDS symptoms (those with opportunistic infections), or coinfections such as tuberculosis or chronic viral hepatitis.

For the following criteria, exclusion occurred when patients concomitantly reported two of them or more: regular performance of intense physical exercise; use of antibiotics, anxiolytics, or antidepressants; active smoking. People with only one of these conditions were included because the statistical analysis was adjusted for these variables.

### 2.3. Sociodemographic and Clinical Data

Sociodemographic and clinical data were collected by interviews and from the patients' medical records, taking into account the date of blood collection for this study.

### 2.4. Analyses of Oxidative Stress Parameters

Twelve milliliters of blood was collected into an EDTA containing tube from each patient included in the study. The material was maintained in a cooled and dark environment for 2-3 hours. After that, 60 *μ*L of total blood was separated for the comet assay procedure and the remaining sample was centrifuged at 1,500 rpm for 10 minutes. Six plasma aliquots per individual were stored at −80°C until the tests were performed. We analyzed the following variables: lipid peroxidation (MDA and 8-isoprostane), DNA damage, TAC and vitamin profile with dosages of carotenoids (lutein, cryptoxanthin, *β*-carotene, and lycopene), retinol, and *α*-tocopherol.

*(i) Measurement of Lipid Peroxidation by Analysis*. The high performance liquid chromatography (HPLC) technique was performed by using the* Shimadzu* HPLC System according to the Nielsen et al. method [23], with 100 *μ*L of plasma. For quantification of chromatograms, a comparison between the substance and the pattern area was made, and results were expressed by *μ*mol/L. 

*(ii) Evaluation of Lipid Peroxidation by Analysis of 8-Isoprostane*. The immunoenzymatic assay (ELISA) was performed according to the manufacturer's instructions (*Cayman*, item 516351) using 50 *μ*L of plasma. Optical densities were calculated in pg/mL. 

*(iii) Analysis of DNA Damage in Peripheral Blood Mononuclear Cells by Comet Assay*. It was developed according to Sasaki et al. [24] using 30 *μ*L of whole blood set on a blade with agarose. After lysis solution treatment, triplicates of the blades were subjected to three different conditions in electrophoresis: basal condition (BAS), formamidopyrimidine DNA glycosylase (FPG), or endonuclease III (END) addition for detection of oxidative damage in purine and pyrimidine bases, respectively* (Biocompare, CA, USA)*. The slides were stained by* Sybr Gold (Invitrogen, USA)*. Using an immunofluorescence microscope connected to an image analysis system* (comet assay IV, Perceptive Instruments, Sufolk, Haverhill, UK)*, a total of 50 randomly selected nucleoids were counted for each slide. Results were expressed as “tail intensity” (ti), which is the percentage of migrated DNA, and “tail moment” (tm), relative values from the fraction of migrated DNA multiplied for the length of the tail. 

*(iv) Analysis of the TAC*. Using 100 *μ*L of plasma, it was determined calorimetrically in triplicate according to the methodology described by Beretta et al. [25] using the* VICTOR X2* reader* (Perkin Elmer, Boston, MA, USA)*. Results were expressed as % of protection. 

*(v) Fat-Soluble Vitamins*. They were measured from 100 *μ*L of plasma by HPLC* (Waters 2996)*, by a C30 column (150 × 4.6 mm, 3 *μ*m) and according to Ferreira and Matsubara [26]. The wavelength used was 455 nm for carotenoids (lutein, cryptoxanthin, lycopene, and *β*-carotene), 325 nm for retinol, and 290 nm for *α*-tocopherol. The values of the standard solution of the substances were fixed by their molar extinction coefficients expressed in *μ*mol/mL.

### 2.5. Analysis of Results

We used a generalized linear model with Poisson or Negative Binomial distribution for count variables and Gamma Distribution for asymmetric variables or One-way ANOVA followed by Tukey-Kramer post hoc tests for those symmetric data in a repeated measure design testing a “moment versus group” interaction. Pearson correlations were adopted to analyze continuous variables.

After fitting the model, confounding variables (age, sex and tobacco use, practice of intense physical activity, and use of anxiolytics and/or antidepressants) were added in order to verify their influence on the “moment versus group” interaction, as is done in a covariance analysis model.

Significant differences were considered when *p* values were less than or equal to 0.05. All these procedures were performed with help from professionals at the institution's Research Support Office using* SAS for Windows, version 9.2* (PROC MIXED and PROC GENMOD).

This study was approved by the Research Ethics Committees of the Botucatu Medical School (Faculdade de Medicina de Botucatu), protocol number 4327-2012.

## 3. Results

The enrolled individuals was primarily male (60%), aged between 20 and 49 years (34 ± 8.2) and most of the patients were diagnosed HIV-positive less than three years ago. The sociodemographic, clinical, and epidemiological characteristics of the subjects included in the study are shown in Table 1.

About eighty percent of patients started cART with first-line regimens which are composed of two NRTIs and one NNRTI. In the second blood collection (M1), 80% of the participants had undetectable viral load.

The evaluation of CD4+ T cell counts in M1 showed an increase in relation to M0 (*p* < 0.0001), which was more evident in G1 compared to G2 (respective gain in cell counts: 209.71, versus 109.62 cells/mL).

**Figure 1:**
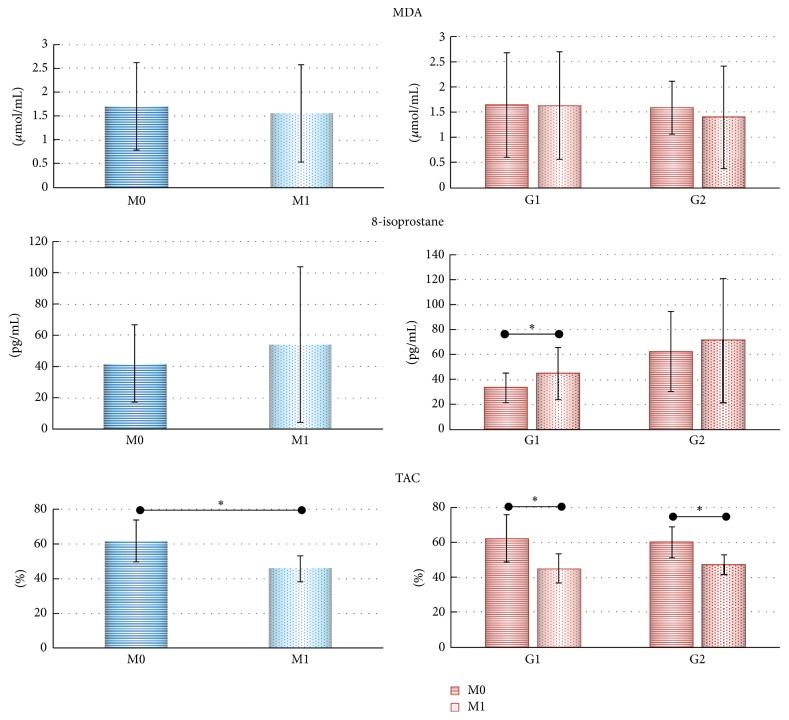
Means of MDA, 8-isoprostane, and TAC of 30 PVWHA before (M0) and after (M1) cART initiation. In blue: patients analyzed together; in red: patients separated into initial CD4+ T groups. G1- CD4+ T counts <500, *n* = 11; G2- CD4+ T >500 cells/mL (considering the counts in M0), *n* = 19. PLWHA: people living with HIV/AIDS; cART: combined antiretroviral therapy; MDA: malondialdehyde; TAC: total antioxidant capacity;* statistical tests*: Gamma Distribution for MDA and 8-isoprostane and ANOVA with post hoc Tukey tests for the TAC. ^*∗*^*p* < 0.05.

No differences were observed in MDA (M0: 1.7 ± 0.9; M1: 1.5 ± 1.0 *μ*mol/mL) or 8-isoprostane levels (M0: 42.0 ± 24.7; M1: 54.2 ± 49.8 pg/mL) after cART initiation; conversely, TAC decreased significantly after cART initiation (M0: 61,8 ± 12.3; M1: 45.7 ± 7.4%; *p* < 0.001). In a separate analysis by groups of G1 and G2 patients, there was no difference in MDA comparing M0 and M1. As for 8-isoprostane G1 subjects showed an increase after cART initiation (*p* = 0.011). For TAC, both groups showed differences between M0 and M1 (G1: *p* < 0.001; G2: *p* = 0.016) (Figure 1).

As for carotenoids, we observed a decrease in lycopene (*p* = 0.015), *β*-carotene (*p* < 0.0001), *α*-tocopherol (*p* = 0.005), and retinol levels (*p* = 0.0021) in M1 compared to M0. After cART we observed a reduction in lutein (*p* = 0.018) and lycopene levels (*p* = 0.019) only in G2 group and in *β*-carotene (*p* < 0.010) and *α*-tocopherol (*p* < 0.050) in both G1 and G2 (Table 2). We also categorized the results of vitamins considering reference values previously established by Kaio et al. [8] (<1.0 for *β*-carotene, <0.7 *μ*mol/L for retinol and <16.0 *μ*mol/L for *α*-tocopherol). The percentages of individuals with values below the ideal, prior to therapeutic initiation, were 93%, 17%, and 60% for *β*-carotene, retinol, and *α*-tocopherol, respectively. These proportions changed to 100%, 7%, and 90% after cART.

**Figure 2:**
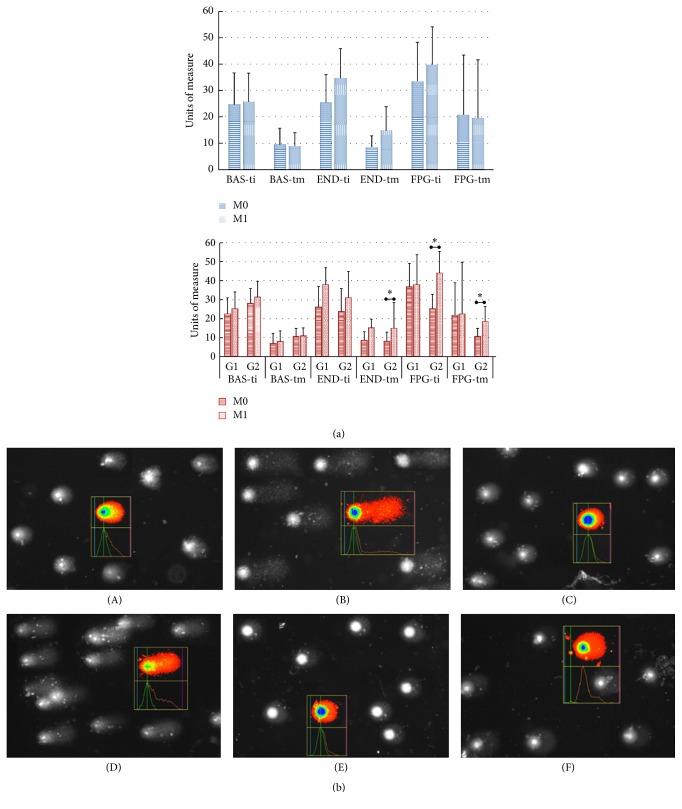
DNA damage of 30 PVHA, before (M0) and after (M1) cART initiation. (a) → In blue: patients analyzed together; in red: patients separated into initial CD4+ T groups. G1- CD4+ T counts <500, *n* = 11; G2- CD4+ T >500 cells/mL (considering the counts in M0), *n* = 19. PLWHA: people living with HIV/AIDS; cART: combined antiretroviral therapy. BAS: basal condition (without enzymes); END: blades treated with the endonuclease III enzyme (thymine glycol -DNA glycosylase); FPG: blades treated with the formanodipirimidina-DNA glycosylase enzyme.* Statistical tests*: Gamma Distribution for Bas-ti and FPG-tm; ANOVA with post hoc Tukey tests for the other variables. ^*∗*^*p* < 0.05.* Units of measure*: ti (tail intensity) and tm (tail moment). (b) → This HIV-infected subject presented initial CD4+ T counts >500 cells/mL, and the figure shows the enzymatic (END [A, B], FPG [C, D]) and basal conditions (BAS [E, F]), before (A, C, E) and after (B, D, F) cART initiation.

In relation to the comet assay, there was a slight increase, although not significant, for the “tail moment” (*p* = 0.068) and “tail intensity” (*p* = 0.097) relative to slides treated with END in M1 compared to M0. However, when analyses were performed considering the G1 and G2 groups, we observed an increase in DNA damage only in G2, at the “tail moment” of the blades treated with FPG (*p* = 0.032) and END (*p* = 0.050), and in the “tail intensity” of the blades treated with END (*p* = 0.012). These data are shown in Figures 2(a) and 2(b).

Additionally, we found a positive correlation between MDA and plasma HIV viral load (VL) (*r* = +0.5858, *p* < 0.001). Negative correlations occurred between retinol and VL (*r* = −0.3821, *p* = 0.033), between *α*-tocopherol and END-ti (*r* = −0.6917, *p* = 0.001) and END-tm (*r* = −0.6182, *p* = 0.006), between lycopene and BAS-tm (*r* = −0.4582, *p* = 0.028) and between END-ti with lutein (*r* = −0.5505, *p* = 0.018), cryptoxanthin (*r* = −0.6480, *p* = 0.004) and *β*-carotene (*r* = −0.5508, *p* = 0.017). No correlation was found between the adopted antiretroviral regimens (PI × NNRTI) and the variables studied in this investigation.

## 4. Discussion

HIV infection is characterized by severe immunodeficiency, a consequence of numerical and functional CD4+ T cell depletion [3]. The patients enrolled in this study showed an increase in CD4+ T cell count after cART initiation (450 versus 625 cells/mL) and 80% of them attained VL undetectability, consistent with an improvement in immune-virological parameters. There is no doubt about the benefits of cART in reducing morbidity and mortality in AIDS [1, 3], but its long-term use is related to many adverse events specially the increase of metabolic disorders and oxidative stress, factors that contribute to the development of non-AIDS comorbidities [4, 5, 19].

It is known that the therapy in some individuals may affect mitochondrial morphology and function [27] and the activation of the P450 cytochrome enzyme system [6], which in turn increases the reactive oxygen (ROS) and nitrogen (RNS) species in circulation. The generation of free radicals as well as the intense consumption and the absence/deficiency of antioxidants are responsible for damaging proteins, cells, and tissues [19]. In addition, cART is associated with metabolic disorders that increase oxidative stress in infected individuals [19]. All of this can lead to the chronic inflammatory status that these patients often present.

Additionally, as demonstrated by our own group in a recent research employing the same cohort of the present study, although there was no difference in the levels of some inflammatory and anti-inflammatory cytokines, there was an increased level of C-reactive protein (CRP) and triglycerides already in the first months of therapy [28]. These markers may also be associated with increased oxidative stress described here in the same period.

Differences in MDA values, a product of lipid peroxidation, were not observed here between pre- and post-cART treatment, nor in relation to initial CD4+ T counts. We just observed a positive correlation between VL and MDA, suggesting the influence of viral pathogenic effects on the increase of oxidative stress. However there are controversial data on this marker in literature. While some authors [29] showed no differences between HIV-infected or uninfected, symptomatic or asymptomatic patients, others [14, 30] have found higher MDA concentration in infected patients with low CD4+ T counts.

Another marker of oxidative stress based on lipid peroxidation is PGF2 alpha-8-isoprostane or 8-isoprostane, the most abundant compound among F2-isoprostanes. We showed no changes in their levels after therapeutic intervention, when patients were analyzed together. However, G1 group showed a significant increase in 8-isoprostanes levels in M1 compared to M0. Hulgan et al. [6] also found elevated plasma concentrations of F2-isoprostane in PLWHA under cART and viral suppression. Crist et al. [31] showed higher 8-isoprostane levels in HIV-infected women as compared to men; therefore it is worth mentioning that, in our study, the analysis was adjusted for gender, age, and other variables that could be confounding.

In relation to the pharmacological treatment, the 16.7% of patients receiving IP/r were taking lopinavir/ritonavir and almost all of the 83.3% of individuals who received NNRTI were taking efavirenz; nevertheless we did not find any correlation between antiretroviral classes and the levels of the analyzed parameters, perhaps due to the short period of therapy using. In this regard, Hulgan et al. [6] and Gupta et al. [32] showed that F2-isoprostane levels increase in subjects under efavirenz as compared to those under rilpivirine, a second-generation NNRTI. Different plasma levels of this marker were also found by Redhage et al. [15] in a drug scheme comparison, showing higher, intermediate, and lower levels in NNRTI-free, treatment-naïve, and NNRTI-treated patients, respectively. High concentrations of F2-isoprostanes were also observed in relation to other chronic diseases and habits, such as atherosclerosis [33], Alzheimer [34], and smoking [35], in HIV-uninfected individuals. Thus, there is need for better understanding this and other markers in the pathogenesis of HIV and non-AIDS-related diseases.

The production of an effective antioxidant system is needed, in order to control this repertory of prooxidants. Human cells are fraught with endogenous and exogenous antioxidants, enzymatic and nonenzymatic [36]. The measurement of TAC considers the cumulative effect of all antioxidants present in blood and body fluids, considering both lipophilic and hydrophilic compartments [25, 37].

In the present study, a decrease in TAC occurred after cART initiation, and these data have been corroborated by other studies [17, 19]. This decrease was independent from the initial CD4+ T cell count; however Suresh et al. [13] showed a lower TAC in HIV-naïve patients than uninfected individuals, and this decrease was more accentuated in those with CD4+ T cells below 500/mL. PLWHA with the metabolic syndrome also showing a decline in TAC, which was independent of the presence of cART [19]. However, the findings by Mandas et al. [17] showed that oxidative stress was higher in patients who adhered to therapy as compared to nonadherents, reinforcing the role of antiretroviral drugs in the induction of these mechanisms.

One of the explanations for low TAC in PLWHA would be the high consumption of antioxidants due to high levels of ROS presented by this population [14]. The other would be the lack of antioxidant intake and of their precursors and/or the poor absorption of nutrients resulting from the changes in intestinal homeostasis [14, 38]. Aspects related to dietary surveys and clinical manifestations suggestive of the malabsorption syndrome were not assessed in this study; thus comparisons were not possible. Therefore, it is important to investigate which components of the antioxidant system are deficient in this population.

Carotenoids have been studied over the years not only for their proven pro-vitamin A activity, but also for their capacity to modulate T cell proliferation, Th17 and regulatory T cell activity in the gut, and production of local immunoglobulin A [39]. Furthermore carotenoids were proven to reduce the risk of degenerative diseases, including certain types of cancer and cardiovascular diseases [40]. Herein, after cART initiation we observed a decrease in lutein, *β*-carotene, lycopene, and retinol levels, which was more evident in the G2 group. These results resemble those reported by other authors [18, 41] which found a 98%  *β* carotene deficiency and 4% retinol deficiency in treated PLWHA; indeed in our cohort 100% of patients had disabilities to *β*- carotene, and only 6.6% showed retinol deficiency after eight months of cART initiation (data not shown). Based on these data, we share the conclusions of those authors [18] suggesting that PLWHA would not benefit from vitamin A supplementation, but from *β*-carotene, as its antioxidant effect could be more beneficial for individuals on cART.

The most abundant active compound of vitamin E, *α*-tocopherol, also plays an essential role in antioxidant protection as it prevents propagation of lipid peroxidation and reduces damage caused by free radicals [12]. Furthermore, it is related to the reduction of proinflammatory cytokines [39]. Some studies [13, 42] on naïve HIV-infected individuals have shown that the deficiency of this and other micronutrients occurred more significantly in patients with lower CD4+ T counts. In our study, *α*-tocopherol showed low levels after cART initiation independently from initial CD4+ T cell count.

Another study by Kaio et al. [8] compared *α*-tocopherol levels with antiretroviral classes and showed a decrease of this vitamin in nearly 20% of PLWHA [8]. Conversely in our study approximately 60% and 90% of the PLWHA showed *α*-tocopherol deficiency before and after cART introduction, respectively (data not shown). Nevertheless, the participants in both studies were from the same region and the same Brazilian state; thus it can be assumed that the populations share similar habits such as diet. In an attempt to justify the difference between these results, it is necessary to consider some aspects. In the cross-sectional study by Kaio et al. [8], at the time of enrollment patients have already been using cART for an extended period, for nearly 10 years, and they had received more than one therapeutic scheme, including therapeutic rescue. On the other hand, in our longitudinal study treatment duration was only eight months on average, and most patients were receiving the first scheme composed of NNRTI which could reduce *α*-tocopherol levels. We could also presume that there may be a greater loss/consumption/deficiency of this vitamin in the first months of therapy in relation to its prolonged use.

As a result of this imbalance between pro- and antioxidants, some genotoxic alterations may occur, therefore we decided to analyze the DNA damage by comet assay. When the patients were analyzed together, we could not observe any significant difference between M0 and M1; conversely increased oxidative genotoxic damage was detected in the group with higher CD4+ T cell count, which could be explained at least in part by the toxicity of cART. To our knowledge, there are no studies assessing DNA damage by comet assay in blood of HIV-infected asymptomatic individuals before and after cART. Aukrust et al. [43] reported that DNA glycosylase, a genomic damage repair marker, showed an inverse correlation with VL and increased of CD4+ T cell levels in individuals who used cART for six months. The same authors [43] showed a reduction in 8-oxoG (7,8-dihydro-8-oxoguanine), a marker of oxidative DNA damage, in CD4+ T cells from subjects with cART as compared to naïve individuals.

de Oliveira et al. [44] showed genotoxic effects in the brains of mice chronically treated with efavirenz and suggested that DNA damage was induced by oxidative stress, which could also explain the neuropsychiatric effects of drug use. However, cART is not the only responsible for increasing the frequency of genotoxic damage in PLWHA. The* tat* HIV protein, due to the generation of superoxide anion by mitochondria, induces intracellular ROS production. They, in turn, activate NF-*κ*B, thus increasing HIV transcription [6], genomic instability, and deficiency in DNA repair, which results in higher mutation rate favoring the onset of cancer [12].

We have to consider some limitations in our study such as a small sample size and, as this was not an interventional randomized trial, it is possible to establish only associations but not cause-effect relationships between variables. Besides that, although our aim was to check the marker's changes in the therapeutic beginning, the short cART duration (only eight months) could not reflect the effects of long-term therapy use and the variability of cART duration among the participants (30–48 weeks) could be one of the factors for the few findings in this study compared to we expected. Other biases are related to the absence of dietary surveys or anthropometric measurements in our patients, which could be interesting mainly for analysis of healthy food consumption and dosages of vitamins related here; the lack of a control group, which would allow us to compare the baseline levels of the parameters chosen in non-HIV-infected individuals; and the TAC biomarker choice, because it is a very nonspecific antioxidant index, which does not refer to any specific pathway. Despite these limitations the studied population underwent rigorous exclusion criteria in order to minimize possible confounding effects in the results.

Our next step is to follow this cohort and analyze these parameters in the future to investigate whether this initial oxidative stress would be altered according to years of cART administration.

## 5. Conclusion

On the whole our results suggest that increased oxidative stress results not only from a direct viral involvement but also from cART administration, which considerably increases such stress in infected asymptomatic HIV-infected individuals already in the first months of therapy.

Indeed, cART seems to increase the production of some prooxidants, such as 8-isoprostane and probably others not measured here, which would diminish TAC, including the carotenoids and vitamin profile. This disturbance in turn would contribute to an imbalance between oxidants and antioxidants and, consequently, to a more sustained genotoxic damage in treated patients, mainly in those with high values of CD4+ T pretherapy (>500 cells/mL).

Therefore, despite the evident benefits of treatment, there is a need for further studies with larger cohorts and more discussion about the best time to begin cART, especially for HIV-infected individuals who are long-term nonprogressors, and those uninfected that choose to take the preexposure prophylaxis. Studies proposing dietary interventions and/or nutrition education, could also contribute to the reduction of oxidative stress and the consequent persistent inflammation.

## Figures and Tables

**Table 1:** Characterization of 30 PLWHA studied.

Variables	MEAN (±SD) or %		
	General	M0	M1
Age (years)	34 (±8.2)	—	—
Male gender	60.0%	—	—
Smokers^#^	30.0%	—	—
Physical activities practice^#^	23.3%	—	—
Use of anxiolytics or antidepressants^#^	6.6%	—	—
Time since HIV diagnosis (years)	2.2 (±3.2)	—	—
post-cART follow up period (months)	8.1 (±1.4)		
cART regimen: NRTI + NNRTI	83.3%	—	—
cART regimen: NRTI + PI/r	16.7%		
CD4+ T nadir (cells/mL)	335.7 (±211.3)	—	—
% of patients presenting CD4+ T <500 cells/mL	—	70.0%	86.6%
CD4+ T counts (cells/mL)	—	454.69 (±136.4)	625.07 (±276.9)^*∗*^
Undetectable HIV viral load	—	3,3%	80.0%^*∗*^

SD: standard deviation; PLWHA: people living with HIV/AIDS; cART: combined antiretroviral therapy; M0: before cART initiation; M1: after cART initiation; NRTI: nucleoside reverse-transcriptase inhibitors; NNRTI: nonnucleoside reverse-transcriptase inhibitors; PI/r: protease inhibitors reinforced with ritonavir; # isolated presence of these cited factors (which were not excluded, due the adjustment of statistical analysis fulfilled). *Statistical tests*: Gamma Distribution and ANOVA, ^*∗*^*p* < 0,001.

**Table 2:** Vitamin profile of 30 PLWHA before (M0) and after (M1) cART initiation.

Variables	M0 (MEAN ± SD)	M1 (MEAN ± SD)	*p* value
Lutein (*μ*mol/mL)^#^	0.06 ± 0.06	0.05 ± 0.04	Ns
G1	0.06 ± 0.06	0.05 ± 0.04	Ns
G2	0.06 ± 0.04	0.04 ± 0.03	0.018

Cryptoxanthin (*μ*mol/mL)^#^	0.08 ± 0.09	0.07 ± 0.07	Ns
G1	0.07 ± 0.05	0.07 ± 0.08	Ns
G2	0.15 ± 0.14	0.07 ± 0.05	Ns

*β*-Carotene (*μ*mol/mL)^#^	0.31 ± 0.39	0.17 ± 0.12	<0.0001
G1	0.28 ± 0.36	0.15 ± 0.10	0.002
G2	0.31 ± 0.44	0.18 ± 0.17	0.001

Lycopene (*μ*mol/mL)^#^	0.66 ± 0.47	0.44 ± 0.24	0.015
G1	1.94 ± 3.98	0.45 ± 0.22	Ns
G2	0.86 ± 0.58	0.32 ± 0.26	0.019

*α*-Tocopherol (*μ*mol/mL)^#^	17.94 ± 8.37	4.14 ± 5.72	0.005
G1	15.82 ± 8.52	4.29 ± 5.85	0.030
G2	18.78 ± 10.53	3.85 ± 6.19	0.048

Retinol (*μ*mol/mL)^#^	2.83 ± 1.42	1.82 ± 0.49	0.002
G1	2.47 ± 1.41	1.86 ± 0.51	Ns
G2	2.78 ± 1.75	1.36 ± 0.48	Ns

PLWHA: people living with HIV/AIDS; cART: combined antiretroviral therapy; G1- CD4+ T counts <500, *n* = 11; G2- CD4+ T >500 cells/mL (considering the counts in M0), *n* = 19; SD: standard deviation; Ns: no significant values;  #  analysis of the 30 total patients without division into groups. *Statistical tests*: ANOVA with post hoc Tukey tests for retinol and Gamma Distribution for the other variables.
